# Inactivation of Polymicrobial Biofilms of Foodborne Pathogens Using Epsilon Poly-L-Lysin Conjugated Chitosan Nanoparticles

**DOI:** 10.3390/foods11040569

**Published:** 2022-02-16

**Authors:** Xingjian Bai, Luping Xu, Atul Kumar Singh, Xiaoling Qiu, Mai Liu, Ahmed Abuzeid, Talaat El-Khateib, Arun K. Bhunia

**Affiliations:** 1 Molecular Food Microbiology Laboratory, Department of Food Science, Purdue University, West Lafayette, IN 47907, USA; bai16@purdue.edu (X.B.); xu653@purdue.edu (L.X.); akstech@gmail.com (A.K.S.); kenny.qiu@wur.nl (X.Q.); mliu86@jh.edu (M.L.); 2 Purdue Institute of Inflammation, Immunology and Infectious Disease, Purdue University, West Lafayette, IN 47907, USA; 3 Clear Labs, San Carlos, CA 94070, USA; 4 Department of Food Hygiene, Assiut University, Assiut 71515, Egypt; ahmedabuzeid95@gmail.com (A.A.); talaatkhateib52@gmail.com (T.E.-K.); 5 Animal Health Research Institute, Agriculture Research Center, Giza, Cairo 12618, Egypt; 6 Department of Comparative Pathobiology, Purdue University, West Lafayette, IN 47907, USA

**Keywords:** biofilm, multi-pathogen, chitosan nanoparticle, ε-poly-L-lysine, inactivation, *Listeria*, *Salmonella*, *E. coli*, *S. aureus*, *Pseudomonas*

## Abstract

A mixed culture (polymicrobial) biofilm provides a favorable environment for pathogens to persist in the food processing environment and to contaminate food products. Inactivation and eradication of such biofilms from food processing environments are achieved by using harsh disinfectants, but their toxicity and environmentally hostile characteristics are unsustainable. This study aims to use food-grade natural nanoparticulated antimicrobials to control mixed-culture biofilms. Chitosan, a natural broad-spectrum antimicrobial biopolymer (polysaccharide) from crustaceans, was derivatized to produce chitosan nanoparticles (ChNP) as a carrier for another broad-spectrum antimicrobial agent, ε-poly-L-lysine (PL), to synthesize ChNP-PL conjugate. The antimicrobial activity of ChNP and ChNP-PL was tested against mixed-culture biofilms. ChNP-PL (~100 nm) exhibited a synergistic antimicrobial and anti-biofilm effect against mono or mixed-culture biofilms of five foodborne pathogens, including *Listeria monocytogenes*, *Staphylococcus aureus*, *Salmonella enterica* serovar Enteritidis, *Escherichia coli* O157:H7, and *Pseudomonas aeruginosa*. ChNP-PL treatment prevented biofilm formation by mono or mixed cultures of *L. monocytogenes*, *P. aeruginosa*, and *E. coli* O157:H7, and bacterial counts were either below the detection limit or caused 3.5–5 log reduction. ChNP-PL also inactivated preformed biofilms. In monoculture biofilm, ChNP-PL treatment reduced *L. monocytogenes* counts by 4.5 logs, *S.* Enteritidis by 2 logs, *E. coli* by 2 logs, and *S. aureus* by 0.5 logs, while ChNP-PL had no inhibitory effect on *P. aeruginosa*. In vitro mammalian cell-based cytotoxicity analysis confirmed ChNP-PL to have no deleterious effect on intestinal HCT-8 cell line. In conclusion, our results show ChNP-PL has strong potential to prevent the formation or inactivation of preformed polymicrobial biofilms of foodborne pathogens.

## 1. Introduction

A major public health concern for the food industry is foodborne illnesses, hospitalizations, loss of lives, and the associated recalls of food products leading to huge financial losses. Globally, foodborne pathogens are responsible for two billion illnesses and over one million deaths annually [1], while in the US, about 48 million illnesses, 128,000 hospitalizations, and 3000 deaths happen annually with an estimated economic burden of about 78 billion dollars [2,3].

The persistence of pathogens in food processing facilities has been considered the single most critical factor in product contamination [4]. Persistence is facilitated by biofilm formation by mono or mixed cultures [5,6,7]. In a sessile physiological state, pathogens and commensals have an increased capacity to form biofilms that are more recalcitrant to antimicrobials compared to the suspension or planktonic cells [8,9]. Biofilm formation is aided by several microbial factors, including multiple virulence factors (fimbriae, curli, flagella, adhesion proteins, and capsules) with redundant functions [10,11]. From raw or undercooked food materials, pathogens find a harborage site or niche in food production facilities or product surfaces and form biofilms [9,12,13], which then serve as a source for foodborne outbreaks, especially in cafeterias, hospitals, cruise ships, and commercial food processing facilities [14].

Metaphorically speaking, biofilm can be described as a “house” that has a structure made up of bacteria-made extracellular polymeric substance (EPS) protecting bacterial cells living inside from harsh extraneous conditions [15]. Biofilm formation is also a strategy for microbes to expand their habitat and colonize new biotic or abiotic surfaces. Therefore, once bacteria are transmitted into food processing facilities, hospitals, cafeterias, or cruise ships through raw foods, they could attach to surfaces and start forming biofilms, which can become a consistent contamination source due to inadequate sanitation. For instance, *Listeria monocytogenes* strains with the same pulsotypes were isolated multiple times from the same food processing environment throughout a year, suggesting some pathogens are capable of escaping or surviving routine sanitation regimens and could recurrently contaminate food products [5]. Similarly, *Salmonella enterica* form biofilm and is also repeatedly isolated from various establishments [16]. Here, we aimed to use two natural antimicrobials to control biofilm formation by *L. monocytogenes*, *Staphylococcus aureus*, *Salmonella enterica* serovar Enteritidis (*S*. Enteritidis), *Escherichia coli* O157:H7, and *Pseudomonas aeruginosa*. 

Biofilms in nature have also consisted of bacterial communities with great diversity instead of single culture biofilm [17]. Some reported that mixed culture biofilms can provide better protection than monoculture. A nosocomial *Bacillus subtilis* isolate was tested to be resistant to peracetic acid because its biofilm prevents the penetration of the biocide [18]. When a peracetic acid-sensitive *S. aureus* strain forms mixed culture biofilm with the *B. subtilis* isolate, the former cells were also protected by the biofilm produced by the latter. Another study also showed that *L. monocytogenes* and *Lactobacillus plantarum* in their mixed culture biofilms were most resistant to 15-min treatment with benzalkonium chloride or peracetic acid than the bacteria in their monoculture biofilms [19]. 

Innovative nontoxic anti-biofilm agents and anti-biofilm surface coating technologies for food processing environments have been developed recently [20,21,22]. A composite film made with a biodegradable polymer, chitosan, included multiple broad-spectrum antimicrobial compounds and has the potential to be applied as a food packaging material [23]. Furthermore, chemical sanitizers are routinely used in the food industry, but their toxicity, potential for carry-over to finished products, and environmentally hostile characteristics make them unfit [24,25]. Nanotechnology-based antimicrobial approaches have been used; however, the uncertainty of food contamination and potential for toxicity limits their application [26,27,28]. Thus, food-grade safe alternative approaches are sought. Chitosan, a natural biopolymer from crustaceans (shrimp, crab), is a polysaccharide and has no known negative health effects; therefore, it has been proposed as an effective alternative bioactive polymer in the food industry [29]. It is a polycationic polymer and possesses a broad-spectrum antimicrobial effect at a certain molecular configuration against both Gram-positive and Gram-negative bacteria. 

Chitosan is an inexpensive, nontoxic polycationic natural biopolymer industrially produced by alkaline (40%–50% NaOH) deacetylation of chitin from mushroom stem [30] shrimp, and crab shells [31]. It is a technologically important and ubiquitous polysaccharide biopolymer and contains more than 5000 glucosamine units (*N*-acetyl glucosamine polymer). Previous studies have reported that the binding of chitosan to cell wall teichoic acids, followed by a potential extraction of membrane lipids, leads to bacterial inactivation [32]. Furthermore, the low molecular weight chitosan nanoparticles showed a stronger antimicrobial effect on biofilm than the high molecular weight nanoparticles [33]. In clinical applications, chitosan also has been evaluated for delivering drugs or pharmaceuticals [34].

Nanoparticles with an overall dimension of <100 nm are shown to have properties that are desirable for the delivery of antimicrobial agents, drugs, functional bioactive molecules in the field of medicine, agriculture, and food [35]. Though the antimicrobial activity of ChNP against certain bacterial species is reported [36,37], knowledge on their effectiveness against preformed biofilms or prevention of biofilms of mixed culture pathogens is limited, which was addressed in this study. Furthermore, we also explored whether the antibiofilm activity of ChNP could be augmented with the addition of another broad-spectrum food-grade antimicrobial peptide, such as ε-poly-L-lysine [38]. ɛ-poly-L-lysine (PL) is a water-soluble, biodegradable, edible, and nontoxic homo-poly-amino acids (25–35 lysine residues, 2.85–3.98 kDa), linked by the peptide bond between the carboxyl and ɛ-amino groups of L-lysine [39]. It is produced by *Streptomyces albulus* and is inhibitory against both Gram-positive and Gram-negative bacteria, yeast, and fungi [40]. PL has been generally recognized as safe (GRAS) by the FDA at levels of up to 50 mg/kg in food (GRAS No. 000135). A recent study showed that the combination of both Ch and PL as a coating on Pacific white shrimp is more effective in extending the shelf life than using each individually [41]. Another study also reported that a combination of Ch, PL, and nisin can effectively inhibit the white blush of fresh-cut carrots [42]. However, the effectiveness of ChNP with PL conjugates (ChNP-PL) on the inactivation of multi-pathogen biofilms is not known. 

In this study, we investigated first, the dynamics of biofilm formation of mono and mixed cultures of *Listeria monocytogenes*, *Staphylococcus aureus*, *Salmonella enterica* serovar Enteritidis, *Escherichia coli* O157:H7 and *Pseudomonas aeruginosa*, and second, the antimicrobial effect of ChNP-PL to prevent biofilm formation or inactivate preformed biofilms of mono or mixed cultures of above pathogens. Though *Pseudomonas aeruginosa* is not a typical foodborne pathogen, as a strong biofilm former, it is known to provide shelter to other pathogens to form polymicrobial biofilms [43,44] thus was used in the study. Our results show both *S. aureus* and *P. aeruginosa* promoted biofilm formation by *L. monocytogenes* while *S.* Enteritidis and *E. coli* suppressed *L. monocytogenes* biofilm. Furthermore, ChNP-PL (~100 nm) was inhibitory against both mono and mixed-culture biofilms; especially it was more effective in preventing biofilm formation than inactivating preformed biofilms.

## 2. Materials and Methods

### 2.1. Bacterial and Mammalian Cell Lines Used in This Study

The bacterial cultures used in this study are listed in Table 1. Before experiments, bacteria from our frozen stocks at −80 °C were inoculated in Tryptic Soy Broth supplemented with 0.6% yeast extract (TSBYE; Becton Dickinson, Franklin Lakes, NJ, USA) and incubated at 37 °C for 18–24 h. A human ileocecal cell line, HCT-8 (ATCC, Manassas, VA, USA), was used for assessing the cytotoxicity of antimicrobial components. HCT-8 cells were recovered from frozen stocks in liquid nitrogen and seeded in T-25 flasks (TPP, Trasadingen, Switzerland) with high glucose Dulbecco’s modified Eagle’s medium (DMEM; HyClone, Logan, UT, USA) supplemented with 10% (*v*/*v*) fetal bovine serum (Atlanta Biologicals, Flowery Branch, GA, USA). HCT-8 cells were maintained at 37 °C with 7% CO_2_ and 95% relative humidity. Medium in the T-25 flasks was changed every three days until about 95% confluence; then, the cell monolayers were trypsinized, counted, and seeded in microtiter plates for experiments. 

### 2.2. Synthesis of Chitosan Nanoparticles and Conjugation with ε-poly-L-lysine 

Chitosan (0.1% or 1 mg/mL; low molecular weight, Sigma-Aldrich, Saint Louis, MO, USA) dissolved in an aqueous solution of acetic acid (1% *v*/*v*) in deionized (DI) water was adjusted to pH 4.6 with NaOH and stored in an autoclaved glass bottle at 4 °C. Solution of sodium tripolyphosphate (1 mg/mL, TPP; Thermo Fisher Scientific, Waltham, MA, USA) in DI water was also added with 1% acetic acid, adjusted to pH 4.6, filter sterilized through a 0.22 µm nitrocellulose filter membrane (Fisherbrand, Waltham, MA, USA), and stored in the same conditions as chitosan solution. ChNP was synthesized using the ionic gelation method [33,45] with modifications. A sterile petri dish containing 15 mL of chitosan solution and a magnetic stir bar was placed on a magnetic stirrer (Thermolyne Cimarec, ALT, East Lyme, CT, USA) operating at level 8. TPP solution (5 mL) was mixed slowly (one drop/25 s) to obtain a final weight ratio of chitosan and TPP to 3:1. The ChNP solution was stirred for another 30 min and then transferred into a 50 mL conical tube (Fisherbrand) on ice and sonicated (Branson Sonifier, Thermo Fisher) for 10 cycles of 30 s with 30 s break between cycles. Then, ChNP solution was filtered through a 0.45 µm membrane filter. To conjugate ε-poly-L-lysine (PL) to the ChNP, TPP solution supplemented with 1% PL was used and filtered through a 0.45 µm. To remove free unbound PL, the samples were passed through a 30 kDa cut-off membrane (Amicon Ultra-15, Millipore Sigma, Burlington, MA, USA), and the retentate containing ChNP-PL was reconstituted with DI water to the same volume before ultrafiltration. The pH of the solution was adjusted to 4.6 using HCl. The size of nanoparticles was measured using Malvern Zetasizer (Malvern, UK).

### 2.3. Antibacterial Activity Testing of Chitosan Nanoparticles

Bacterial inhibition zone tests were carried out on brain-heart infusion (BHI; Thermo Scientific, Frederick, MD, USA) soft agar plates, which were prepared by dissolving vendor-suggested amount of BHI medium and 0.8% (*w*/*v*) agar in DI water and autoclaved. After cooling down the soft agar in a 50 °C water bath, 30 mL of the soft agar was transferred into a 50 mL sterile conical tube (Fisherbrand) and kept at ambient temperature for approximately 3 min. Then, 10 μL of fresh overnight bacteria cultures grown in BHI at 37 °C was added into the tube, mixed, and poured into a sterile round petri dish (10 cm × 10 cm, Fisherbrand). Wells were dug on the solidified agar using a cork borer and filled with 10 μL non-solidified soft agar to seal the bottom. Eight microliters of test samples were dispensed into the wells, and the plates were incubated at 37 °C for 24 h.

To specifically quantify the minimal inhibition concentration (MIC) of samples, we adopted the method described before [46]. Briefly, bacterial cultures were incubated in BHI at 37 °C for overnight and diluted in 2× Mueller Hinton Broth (MHB, Beckton Dickinson). One hundred microliters of MHB containing approximately 10^3^ CFU/mL bacteria were added to each well on a 96-well microtiter plate (TPP). Serially diluted antibacterial substances and sterile DI water were added into each well to make up to 200 μL. Bacterial growth was determined by measuring the turbidity of the well content using a spectrophotometer (BioTek, Winooski, VT, USA) at wavelength 595 nm.

### 2.4. Cell Proliferation and Cytotoxicity Tests of ChNP-PL

HCT-8 cells (ATCC, Manassa, VA, USA) cultured in high glucose DMEM (HyClone, Logan, UT, USA) supplemented with 10% fetal bovine serum (D10F; Atlanta Biologicals) were trypsinized (HyClone) and seeded into tissue culture treated 96-well microtiter plates (TPP, Switzerland). The cells were incubated at 37 °C with 7% CO_2_ and 95% relative humidity for a week. Before the experiment, cells from three wells were detached by trypsinization and counted using a hemocytometer. Cell growth medium was replaced with 100 μL fresh D10F containing 10 μL ChNP-PL and/or fresh *L. monocytogenes* F4244 at a multiplicity of infection (MOI) of 10. Untreated cells and cells only treated with *L. monocytogenes* were used as negative and positive controls, respectively. After incubating cells under the previously-mentioned conditions for 13 h, 10 μL of WST-8 (water-soluble tetrazolium-8) substrate (Millipore Sigma) was directly added to cells for proliferation assay. Then cells were further incubated under the same conditions for another 2 h, and the optical density of the wells was measured at 450 nm using a spectrophotometer. For cytotoxicity assay, 100 μL of cell supernatant after the 13 h incubation was collected and subjected to lactate dehydrogenase (LDH) assay (Thermo Fisher Scientific) following the vendor’s instruction. Measurements from the supernatant of cells lysed by 0.1% Triton-X and untreated cells were used for percent cytotoxicity calculations [28]. 

### 2.5. Single and Mixed Culture Biofilm Formation

Bacteria cultures (*L. monocytogenes* F4244, *S. aureus* ATCC25923, *P. aeruginosa* PRI99, *S.* Enteritidis 18ENT1344, and *E. coli* EDL933) were recovered from frozen stocks in −80 °C, inoculated into tryptic soy broth (TSB), and incubated under 37 °C for 24 h. The optical density (OD_595nm_) of cultures was adjusted to 1.2 (~1–5 × 10^9^ CFU/mL) and then diluted (1:200) in TSB (45 mL). Then, the cultures were transferred to a tissue culture-treated petri dish (TPP, Switzerland) to provide enough surface area for biofilm formation at 30 °C for 24 h [47,48]. To disrupt biofilms, media were removed, and biofilms were washed once with 5 mL sterile PBS to remove loosely attached cells. Another 5 mL PBS was added to the biofilms, and the Petri dishes were sonicated for 15 min in a cold-water bath sonicator (iSonic, Chicago, IL, USA). To detach strong biofilms produced by *S. aureus* ATCC25923 or *P. aeruginosa* PRI99, a sterile cell scraper was used to manually scrape the cells from the bottom of the Petri dish. The bacteria in PBS was further diluted and plated on BHI agar and Modified Oxford medium (MOX) agar plates for enumeration. The *Listeria* counts from MOX plates were subtracted from the total counts in BHI to estimate the partner cell counts. 

### 2.6. Prevention of Biofilm Formation and Inactivation of Preformed Biofilm by ChNP-PL

Freshly grown (at 37 °C for 18 h) bacterial cultures were diluted to about 10^3^ CFU/mL in TSB, and 800 μL of the diluted culture suspension was dispensed into wells of 24-well tissue culture plates (TPP, Switzerland). For mixed culture biofilms, 800 μL of each culture containing about 10^3^ CFU/mL was added per well. For the assessment of prevention of biofilm formation, 200 μL of ChNP or ChNP-PL preparation was added to each well and incubated at 30 °C for 24 h. Thereafter, the TSB medium was removed, and each well was rinsed twice with PBS (500 μL) to remove loosely attached bacteria. To disrupt biofilm, 200 μL PBS was added to each well, sealed with parafilm, and sonicated for 15 min in a water bath sonicator (iSonic). Samples were diluted in PBS and plated on BHI or MOX agar plates. In addition, biofilms in the well were stained by crystal violet (CV) staining [46,49,50]. Briefly, 96-well tissue culture plates were seeded with 200 µL of TSB and 50 µL of antimicrobial substance. After 24 h incubation at 30 °C, biofilms were rinsed twice with 100 μL PBS, air-dried for 15 min, and stained with 200 μL 0.1% CV solution for 45 min at room temperature. Excess CV solution was removed, and wells were washed twice with 100 μL PBS and photographed. 

For the inactivation of preformed biofilms, bacteria were inoculated in 24-well tissue culture plates and incubated at 30 °C for 24 h. Next, wells were gently rinsed once with PBS (500 μL) to remove loosely attached bacteria. Then, 800 μL MHB containing 200 μL of ChNP-PL or ChNP preparation was added to the wells, and the plates were further incubated at 30 °C for an additional 24 h. Biofilms were gently rinsed twice with PBS as before, and sessile bacteria in biofilms were quantified by plating.

## 3. Results

### 3.1. Synthesis of Chitosan Nanoparticles Conjugated with ε-poly-L-lysine (ChNP-PL)

In acetic acid (1%) solution, chitosan forms a positively charged chain-like structure; thus, negatively charged tripolyphosphate (TPP) is added as an anionic linker to crosslink chitosan molecules by binding to their positively charged amino groups to form chitosan nanoparticles (ChNP) [51]. After testing different combinations of chitosan and TPP, mixing one volume of 0.1% TPP solution into three volumes of 0.1% chitosan solution (final weight ratio of Ch:TPP equals 3:1) generated ChNP with the medium size of 100–200 nm (Figure S1a). After passing the preparation through a 0.45 µm filter, ChNP with uniform size (median = 164 nm) was achieved (Figure S1a,b). Furthermore, the application of a sonication step (10 cycles, 30 s each) before filtration reduced ChNP dimension from about 164 nm to 91 nm (Figure S1c), which were used for further studies. Note, a weight ratio of 4:1 or 6:1 (Ch:TPP) using 0.2% chitosan produced particles that are greater than 1000 nm thus this approach was no longer pursued.

**Figure 1:**
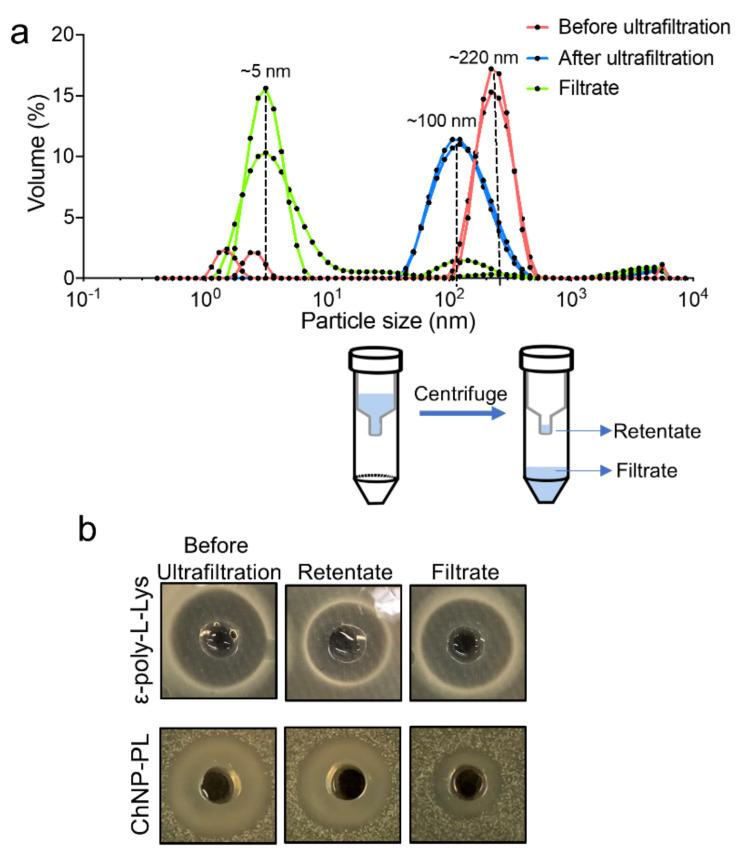
Synthesis and characterization of chitosan nanoparticles (ChNP) and ChNP conjugated to ε-poly-L-Lysine (ChNP-PL). (**a**) Size comparison of ChNP synthesized with or without 1% PL. (Bottom) Removal of unbound PL after ChNP-PL synthesis by ultrafiltration (30 kDa cut-off). (**b**) ChNP-PL and PL-mediated inhibition of *L. monocytogenes* F4244 in soft BHI agar, demonstrating the antimicrobial activity of ChNP-PL.

Next, ε-poly-L-Lysine (PL, 2%) was supplemented in the 0.1% TPP to synthesize ChNP, and the median diameter of the particles was increased from about 96 nm to 370 nm (Figure S1d). However, a sonication step reduced the median size of ChNP-PL to 330 nm (Figure S1d). To further decrease the size of ChNP-PL, we reduced the concentration of PL in TPP from 2% to 1%, which lowered the median size from 330 nm to 220 nm (Figure 1a and Figure S1d). Next, the extra unbound PL was removed by ultrafiltration (30 kDa cut-off membrane) (Figure 1a), and then the median size of ChNP-PL decreased to about 100 nm (Figure 1a). The particles in ultrafiltration filtrate had a median size of about 5 nm, suggesting ChNP cannot pass through the 30 kDa cut-off membrane during ultrafiltration (Figure 1a). 

Antimicrobial activity testing by well diffusion assay against a lawn of *L. monocytogenes* F4244 cells on an agar plate demonstrated that the activity (zone of inhibition) of ChNP-PL in retentate was about 1.96-fold of the activity observed for the filtrate, which consisted of mostly the free PL. The antimicrobial activity of the filtrate was tested and compared with 1% PL solution after passing through the membrane. The activity of PL in the filtrate was observed to be higher than the retentate, thus suggesting that the smaller PL molecules are free to move through the membrane (Figure 1b). While in the ChNP-PL preparation, most of the PL remained bound and were present mostly in the retentate fraction (Figure 1b). According to the correlation function (R^2^ = 0.995) between the PL concentration and the size of inhibition zone on *L. monocytogenes* in the BHI agar plate, it was estimated that about 63.7% of PL remained bound to the ChNP-PL preparation (Figure S1e). 

### 3.2. ChNP and PL Exhibited Synergistic Antimicrobial Activity 

**Figure 2:**
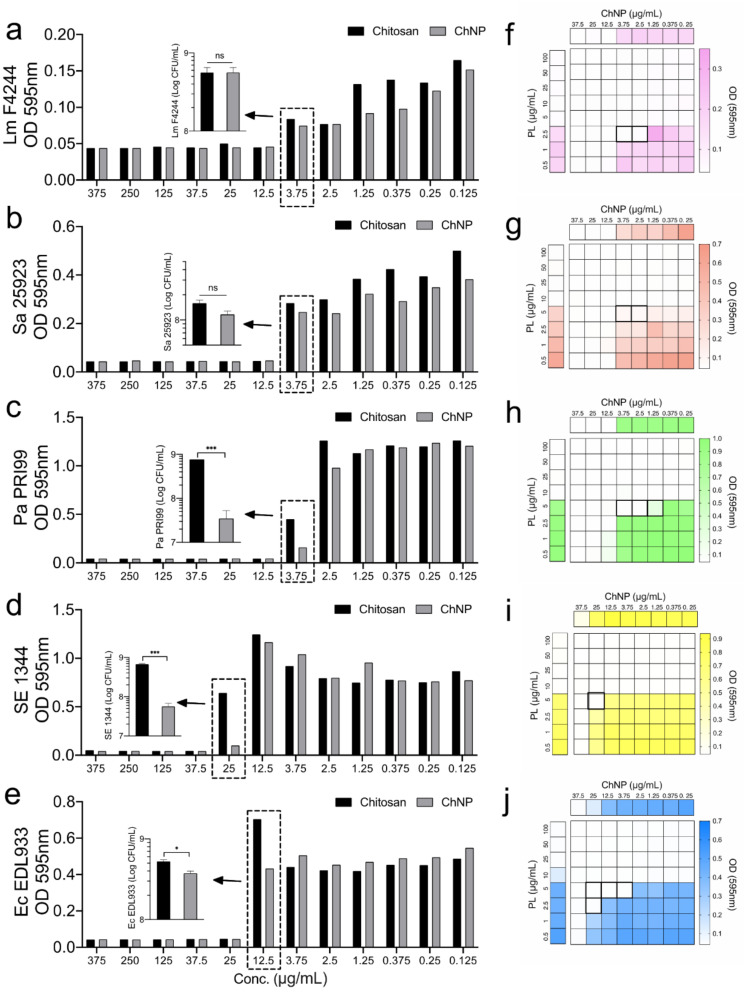
Comparison of the minimal inhibitory concentration (MIC) of chitosan and chitosan nanoparticles (ChNP) on (**a**) *L. monocytogenes* F4244 (Lm), (**b**) *S. aureus* ATCC25923 (Sa), (**c**) *P. aeruginosa* PRI99 (Pa), (**d**) *S.* Enteritidis 13ENT1344 (SE), and (**e**) *E. coli* EDL933 (Ec) in Mueller Hinton Broth (MHB). Synergistic MIC of ChNP and PL on (**f**) *L. monocytogenes* F4244, (**g**) *S. aureus* ATCC25923, (**h**) *P. aeruginosa* PRI99, (**i**) *S.* Enteritidis 13ENT1344, and (**j**) *E. coli* EDL933 was lower than the individual MIC of ChNP or PL. The boxes with bold boundaries indicate the reduced concentration of ChNP and PL when they act synergistically to the pathogens. A pairwise Student’s *t-*test was used for statistical analysis. * *p* < 0.05, *** *p* < 0.0005, ns, non significant.

First, the antimicrobial activity of chitosan polymer and ChNP was compared by using the MIC assay using a microdilution method [28] against five pathogens, *L. monocytogenes* F4244, *S. aureus* ATCC25923, *P. aeruginosa* PRI99, *S.* Enteritidis 13ENT1344, and *E. coli* EDL933 (O157:H7). Bacterial growth inhibition results (OD_595nm_) showed that the MIC of chitosan polymer and ChNP was very similar depending on the strains tested (Figure 2a–e). MIC of chitosan and ChNP against *L. monocytogenes*, *S. aureus*, and *P. aeruginosa* were estimated to be 12.5 μg/mL while and 25 μg/mL for *E. coli* O157:H7. In addition, the MIC of chitosan against *S.* Enteritidis was 37.5 μg/mL while ChNP was 25 μg/mL (Figure 2a–e). Plate counts of *P. aeruginosa*, *S.* Enteritidis, and *E. coli* treated by ChNP were significantly (*p* < 0.05) lower than those bacteria treated by chitosan polymer (Figure 2a–e). 

Next, to test the synergistic antimicrobial effects of ChNP and PL on these pathogens, MIC tests were conducted by adding the decreasing concentration of both samples in each well on a 96-well microtiter plate. Results showed that the MIC of a mixture of ChNP and PL is lower than the MIC of each tested separately, suggesting they possess synergistic antimicrobial effects (Figure 2f–j). 

Next, MIC of ChNP and ChNP-PL was compared using microdilution methods against several pathogenic or nonpathogenic bacteria. Results showed that the MIC of ChNP-PL was lower than ChNP on all the 19 cultures tested (Table 1 and Figure S2). Compared to the MICs of ChNP, MICs of ChNP-PL on *Pseudomonas* (two species) and *Salmonella enterica* (three serovars) were reduced by 3-fold, and *Listeria* (four species), *S. aureus* (two strains), and *E. coli* (three strains) were reduced by 10-fold, suggesting conjugation of ChNP with PL significantly improves its inhibitory effect. 

**Figure 3:**
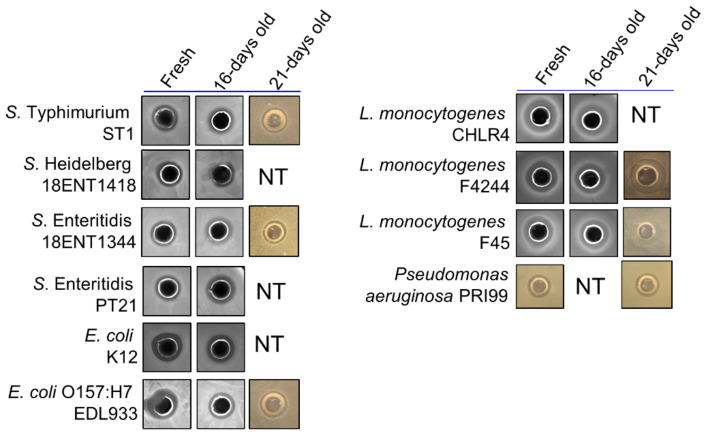
Comparison of the antimicrobial activities (zone of inhibition) of fresh ChNP-PL and ChNP-PL stored at ambient temperature for 16–21 days using an agar gel diffusion method. NT: not tested.

Further, we tested the stability of ChNP-PL stored at ambient temperature for 16–21 days. Both freshly prepared and 16–21-day stored ChNP-PL produced a similar zone of inhibition when tested against lawns of *Salmonella*, *E. coli*, *L. monocytogenes*, and *P. aeruginosa* by well-diffusion method (Figure 3). This suggests the antibacterial activity of ChNP-PL is maintained at least for 16–21 days in ambient conditions.

### 3.3. ChNP-PL Is Nontoxic to Intestinal Epithelial Cells 

**Figure 4:**
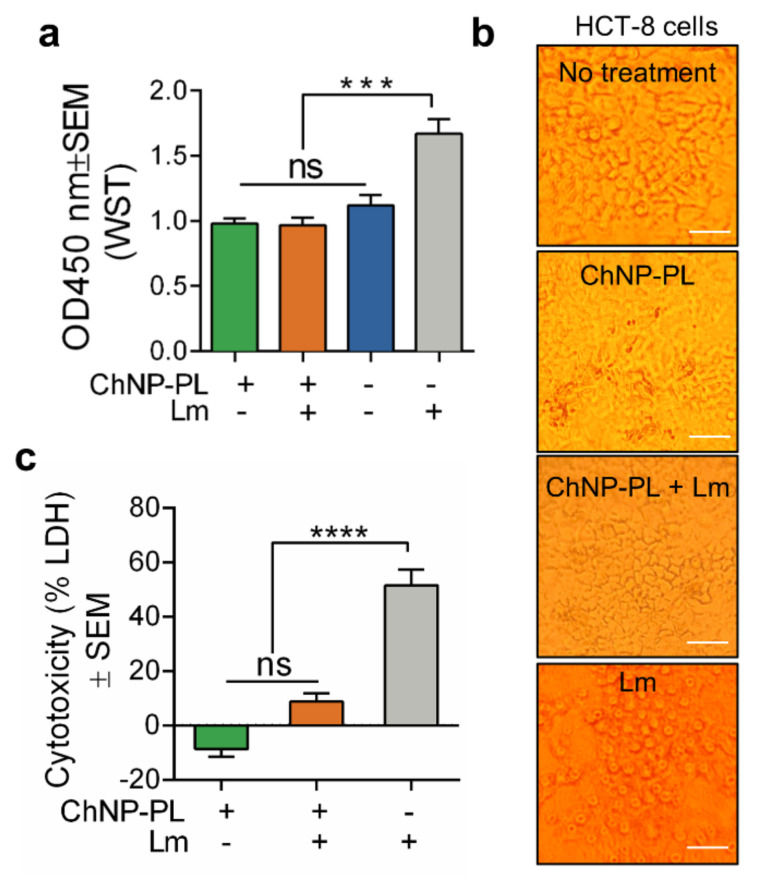
Cytotoxicity assessment of ChNP-PL on HCT-8, an intestinal epithelial cell line, using (**a**,**b**) water-soluble tetrazolium (WST-8) or (**c**) lactate dehydrogenase (LDH) assay. Cells were incubated with or without ChNP-PL (1:10 diluted) and *L. monocytogenes* F4244 (MOI 1:10; Lm:HCT-8) for 13 h and analyzed for cell proliferation or cytotoxicity. (**b**) Microscopic images of cell monolayers after the completion of the WST assay. None of the ChNP preparations caused any cell damage, except the positive control where cell monolayers received only Lm. Scale bars, 50 μm. A pairwise Student’s *t*-test was used for statistical analysis. *** *p* < 0.0005, **** *p* < 0.0001, ns, non significant.

Cytotoxicity effects of ChNP-PL were tested on HCT-8 cell line (human colorectal adenocarcinoma cells), using both WST-8 (2-(2-methoxy-4- nitrophenyl)-3-(4- nitrophenyl)-5-(2,4-disulfophenyl)-tetrazolium) and LDH (lactate dehydrogenase) assays. WST-8 assay measures cell proliferation by reacting with NADH (Nicotinamide adenine dinucleotide hydride) from live cells and generating color formazan dye. After incubating HCT-8 cells with 1:10 diluted ChNP-PL for 13 h and WST-8 substrate for another 2 h, the proliferation of treated cells was not significantly (*p* > 0.05) different from untreated cells (Figure 4a). Furthermore, when *L. monocytogenes* F4244 was added to HCT-8 cells together with ChNP-PL at MOI of 1:10 (bacteria:HCT-8 cells), the proliferation of the HCT-8 cells was not significantly (*p* > 0.05) different from untreated or ChNP-PL-treated cells (Figure 4a). On the other hand, HCT-8 cells treated with only *L. monocytogenes* F4244 at the same MOI showed about 50% increase and significantly (*p* < 0.0005) higher absorbance than cells received the three other treatments (Figure 4a). The increment in reading was indicative of *L. monocytogenes* induced cell damage leading to the release of intracellular enzymes and NADH. 

Microscopic comparison of HCT-8 cell monolayers after WST-8 assay indicated maintenance of cell monolayer integrity when treated with ChNP-PL in the presence or absence of *L. monocytogenes* while cell rounding and the detached monolayer were evident when treated with *L. monocytogenes* alone (Figure 4b). These data indicate ChNP-PL is nontoxic and could protect epithelial cells from *L. monocytogenes*-induced cell damage.

We also verified the ChNP-PL effect on HCT-8 cells using a second cytotoxicity assay that assesses the membrane damage by monitoring the release of lactate dehydrogenase (LDH). After 13 h incubation with ChNP-PL, the cytotoxicity value was below zero while ChNP-PL plus *L. monocytogenes* F4244 treatment produced a cytotoxicity value of 10%, and there was no significant difference (*p* > 0.05) between the two treatments (Figure 4c). While HCT-8 cells treated with *L. monocytogenes* alone for 13 h showed about 50% LDH release, which was significantly (*p* < 0.0001) higher than the values from the other two treatments (Figure 4c). These results suggest that ChNP-PL has little or no cytotoxicity or anti-proliferative effect on HCT-8 cells in 13 h. Furthermore, ChNP-PL can also protect epithelial cells from the damage caused by *L. monocytogenes*. 

### 3.4. Shifting of Population Dynamics within Mixed Culture Biofilms 

**Figure 5:**
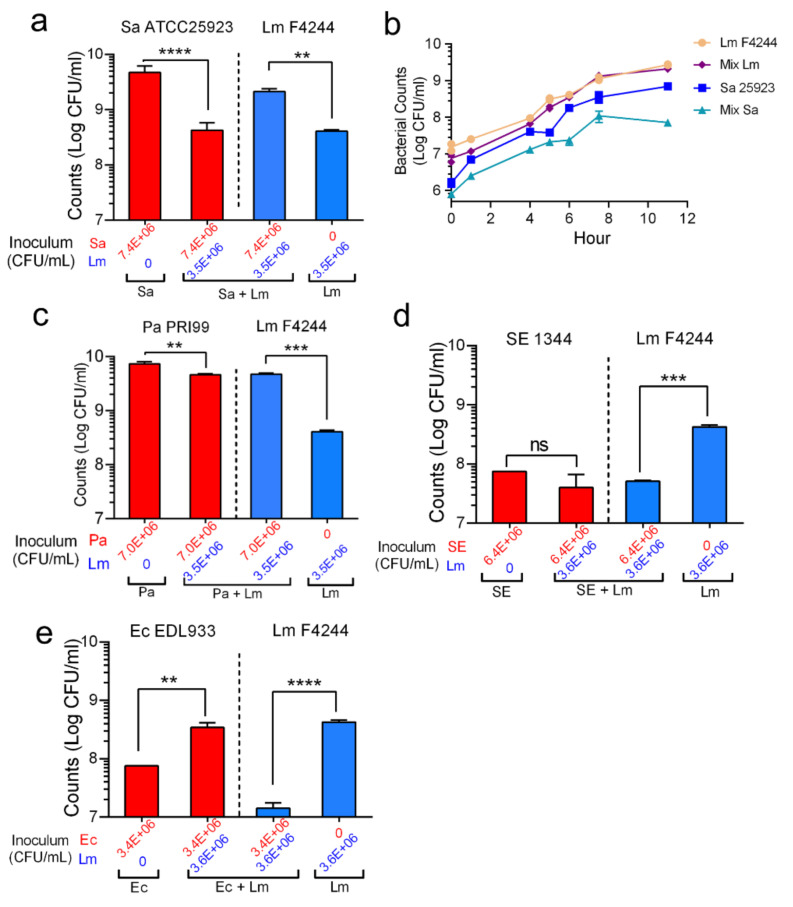
Quantification of bacterial counts in single and mixed culture biofilms. Bar graphs showing counts of (**a**) *S. aureus* ATCC25923 and *L. monocytogenes* F4244, (**b**) growth curve showing mono or mixed culture of *S. aureus* ATCC25923 and *L. monocytogenes* F4244, (**c**) bar graphs of *P. aeruginosa* PRI99 and *L. monocytogenes* F4244, and (**d**) bar graphs of *S*. Enteritidis 18ENT1344 (SE1344) and Lm F4244 (**e**), *E. coli* O157:H7 EDL933 (Ec EDL933) and Lm F4244 in their single or mixed biofilms. A pairwise Student’s *t*-test was used for statistical analysis. ** *p* < 0.005, *** *p* < 0.0005, **** *p* < 0.0001, ns, non significant.

Population dynamics of each bacterial pathogen in a mixed culture biofilm were analyzed. In a mixed culture biofilm of *L. monocytogenes* and *S. aureus*, *L. monocytogenes* counts increased to 2.1 × 10^9^ CFU/mL (a 5-fold increase) when compared with its monoculture biofilm counts (4.1 × 10^8^ CFU/mL) after 24 h of incubation at 30 °C (Figure 5a and Table 2). In contrast, *S. aureus* ATCC25923 counts decreased to 4.2 × 10^8^ CFU/mL (about 10-fold reduction) compared to its count (4.7 × 10^9^ CFU/mL) in monoculture (Figure 5a and Table 2). The observed suppression of *S. aureus* growth in the presence of *L. monocytogenes* was also verified in a growth curve experiment where *L. monocytogenes* growth was not affected while the *S. aureus* growth was (Figure 5d).

In the mixed culture biofilm of *L. monocytogenes* and *P. aeruginosa*, *L. monocytogenes* count was significantly (*p* < 0.0005) higher compared to its count in the monoculture biofilm (Figure 5b). In the mixed culture biofilm with *S.* Enteritidis (Figure 5d) or *E. coli* O157:H7 (Figure 5e) biofilms, *L. monocytogenes* counts were significantly reduced than its monoculture counts. Interestingly, *L. monocytogenes* did not interfere with *S.* Enteritidis growth in the biofilm but augmented *E. coli* growth in the biofilm. Collectively, these data indicate both *S. aureus* and *P. aeruginosa* promoted biofilm formation by *L. monocytogenes* while *S.* Enteritidis and *E. coli* suppressed *L. monocytogenes* growth in their respective mixed cultures biofilms.

### 3.5. ChNP-PL Effectively Prevented Biofilm Formation by Mono- or Mixed-Cultures

**Figure 6:**
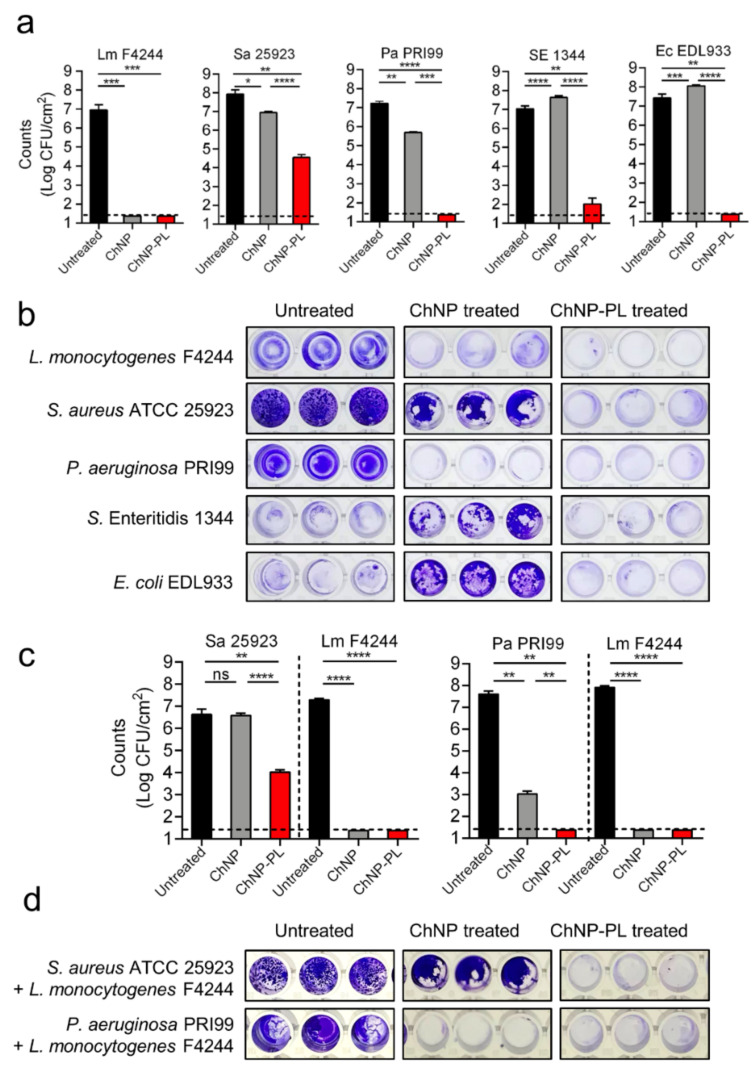
Assessment of the prevention of biofilm formation by (**a**,**b**) monoculture or (**c**,**d**) mixed cultures by chitosan nanoparticles (ChNP) or chitosan nanoparticles conjugated to ɛ-poly-L-lysine (ChNP-PL) on five foodborne pathogens, *L. monocytogenes* (Lm) F4244, *S. aureus* (Sa) 25923, *P. aeruginosa* (Pa) PRI199, *S*. Enteritidis (SE) 1344, and *E. coli* (Ec) O157:H7 EDL933. Panels are (**a**) bacterial counts, (**b**) crystal violet staining of monoculture biofilms in a microtiter plate, and (**c**) bacterial counts and crystal violet staining of mixed culture biofilms of Lm and Sa, or Lm and Pa with ChNP or ChNP-PL. Bacteria isolated from biofilms were quantified by the plating method. A pairwise Student’s *t*-test was used for statistical analysis. * *p* < 0.005, ** *p* < 0.005, *** *p* < 0.0005, **** *p* < 0.0001. Dotted lines represent the detection limit.

We tested the ability of ChNP-PL to prevent monoculture biofilm formation by each of the five pathogens, *L. monocytogenes* F4244, *S. aureus* ATCC25923, *P. aeruginosa* PRI99, *S.* Enteritidis 18ENT1344, and *E. coli* EDL933, and the inhibition data were compared with ChNP-mediated inhibition. Each bacterium inoculated at about 1 × 10^3^ CFU/mL in fresh TSB containing ChNP or ChNP-PL in wells of a 24-well microtiter plate and incubated at 30 °C for 24 h to form biofilms. Then, crystal violet staining and plate counting were used to assess biofilm formation. ChNP-PL treatment prevented biofilm formation by *L. monocytogenes*, *P. aeruginosa*, and *E. coli* O157:H7, and bacterial counts were below the detection limit, while it caused a 5-log reduction in *S.* Enteritidis counts and 3.5 log reduction in *S. aureus* counts (Table 3 and Figure 6a). Though ChNP prevented biofilm formation by *L. monocytogenes*, it showed only 1 log reduction in *S. aureus* counts and about 1.7 log reduction in *P. aeruginosa* counts (Table 3 and Figure 6a). In contrast, it had no inhibitory effect against *S.* Enteritidis or *E. coli*, rather it promoted bacterial growth with about 0.5 log increase in bacterial counts for both (Table 3 and Figure 6a).

Crystal violet staining provided a strong visual corroborating evidence for inhibitory activity of ChNP-PL against all tested organisms (Figure 6b). Untreated control biofilms showed intense dye-binding appearing dark blue, while partially inhibited biofilms showed moderate dye-binding while the wells without biofilms appeared clear. As stated above, ChNP appears to promote biofilm formation by *S*. Enteritidis and *E. coli* O157:H7, showing intense dye-binding after ChNP treatment compared to the untreated controls, which showed partial dye-binding again suggesting ChNP appears to promote biofilm formation by these two pathogens. In contrast, ChNP-PL prevented biofilm formation by these pathogens, and the wells appeared colorless or with a hint of stain (Figure 6b). 

Inhibitory activity of ChNP-PL against the mixed-culture biofilm of *L. monocytogenes* and *P. aeruginosa*, and *L. monocytogenes* and *S. aureus* were examined (Figure 6c). Similar to the monoculture experiment, ChNP-PL completely inhibited the *L. monocytogenes* since the bacterial counts were below the detection limit while it caused about a 3.5-log reduction in *S. aureus* counts. In *L. monocytogenes* and *P. aeruginosa* mixed culture biofilms, ChNP-PL also completely inhibited biofilm formation by both pathogens since the counts were below the detection limit. In these experiments, ChNP abolished *L. monocytogenes* growth and reduced *P. aeruginosa* growth by 4.5 logs; however, ChNP did not show any inhibition of biofilm formation by *S. aureus* (Table 3 and Figure 6c). Crystal violet staining images corroborated with the plate counting data (Table 3 and Figure 6d). These data again demonstrate that ChNP-PL is highly effective in preventing mixed culture biofilm formation by *L. monocytogenes* and *P. aeruginosa* or *L. monocytogenes* and *S. aureus*. Collectively, our data show ChNP-PL is highly effective in preventing single or mixed culture biofilms of five pathogens tested. 

### 3.6. ChNP-PL Inactivated Preformed Biofilms by All Tested Bacteria except P. aeruginosa

**Figure 7:**
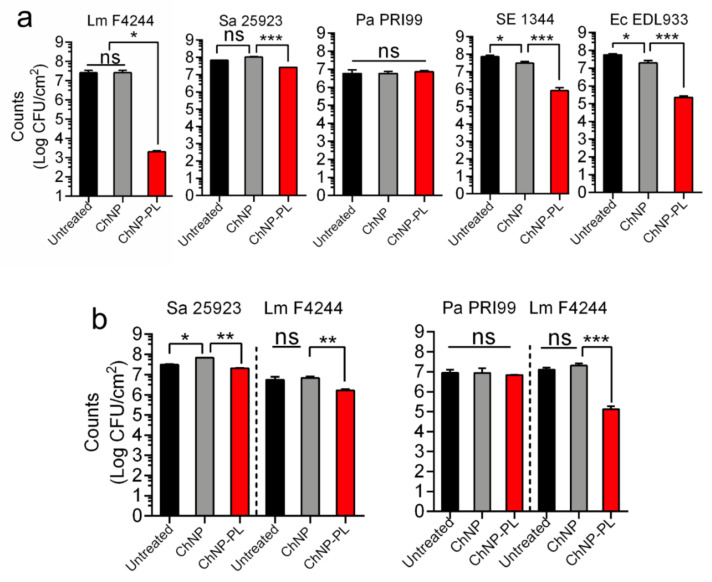
Assessment of inactivation of preformed biofilms of (**a**) monocultures and (**b**) mixed cultures by chitosan nanoparticles (ChNP) or chitosan nanoparticles conjugated to ɛ-poly-L-lysine (ChNP-PL) on five pathogens. Panels are bacterial counts of (**a**) monoculture of *L. monocytogenes* (Lm) F4244, *S. aureus* (Sa) ATCC25923, *P. aeruginosa* (Pa) PRI99, *S*. Enteritidis (SE)18ENT1344, or *E. coli* (Ec) O157:H7 EDL933, and (**b**) mixed culture biofilms of *L. monocytogenes* F4244 and *S. aureus* ATCC25923, or *L. monocytogenes* F4244 and *P. aeruginosa* PRI99. Bacteria isolated from biofilms were quantified by the plating method. A pairwise Student’s *t*-test was used for statistical analysis. * *p* < 0.005, ** *p* < 0.005, *** *p* < 0.0005, ns, non significant.

We tested the ability of ChNP-PL to inactivate/disrupt preformed mono- or multi- pathogen biofilms, and data were compared with ChNP-mediated activity. After pathogens were incubated in wells for 24 h to form biofilms, ChNP-PL or ChNP was diluted by 1:5 (*v/v*) in MHB and added to the wells for another 24 h and incubated at 37 °C. Then, sessile bacterial counts in treated and untreated biofilms were enumerated. In monoculture biofilm, ChNP-PL treatment reduced *L. monocytogenes* F4244 counts by 4.5 logs, *S.* Enteritidis by 2 logs, *E. coli* by 2 logs, and *S. aureus* by 0.5 logs, while ChNP-PL had no inhibitory activity on *P. aeruginosa* (Figure 7a). In contrast, ChNP had no inhibitory effect against *L. monocytogenes*, *S. aureus*, and *P. aeruginosa* but showed a slight inhibitory effect against *S*. Enteritidis and *E. coli* (Figure 7a). These data indicate that ChNP-PL is highly effective in inactivating preformed biofilms though the response was variable depending on the bacterial species tested.

In mixed culture biofilms of *L. monocytogenes* and *S. aureus*, ChNP-PL reduced *L. monocytogenes* counts by 0.3 logs and *S. aureus* by 0.1 log (Figure 7b). In *L. monocytogenes* and *P. aeruginosa* mixed biofilms, ChNP-PL reduced *L. monocytogenes* counts by 2 logs but did not show any inhibitory effect against *P. aeruginosa*. Surprisingly, ChNP did not show any inhibitory effect against none of the pathogens in the mixed culture biofilms (Figure 7).

## 4. Discussion

In this study, we investigated the anti-biofilm activity of two natural antimicrobials (chitosan and ε-poly-L-lysine) to control mono or mixed culture biofilm formation by *L. monocytogenes*, *S. aureus, Salmonella enterica, E. coli* O157:H7, and *P. aeruginosa* because they not only are frequently isolated from the food processing environments but also isolated from the same location [5,44,52,53]. We observed higher *L. monocytogenes* counts in mixed biofilms with *S. aureus* and *P. aeruginosa* than the counts in monoculture biofilms of *L. monocytogenes*. Similarly, Carpentier et al. [54] isolated more *L. monocytogenes* cells in the mixed biofilm with a food plant isolated *Staphylococcus capitis* strain than in the monoculture biofilm. In addition, we also tested mixed biofilm of other bacteria in which *L. monocytogenes* counts decreased in the presence of *S*. Enteritidis and *E. coli* O157:H7. These data indicate bacterial metabolism, and quorum sensing (QS) and 3′,5′-cyclic di-guanosine monophosphate (c-di-GMP) signaling network probably dictate differential bacterial population dynamics within a biofilm [6,55,56,57].

Although various types of antimicrobial disinfectants, like benzalkonium chloride and peracetic acid, have been used in food processing environments [25], they pose a health risk. Peracetic acid is a strong oxidizer that can inactivate microbial enzymes and other functional proteins [58], but personal protection is necessary to prevent eye, respiratory tract, and skin irritations, lethal hemorrhage, and edema [59]. Benzalkonium chloride at a sub-lethal concentration can generate resistant strain as seen in *L. monocytogenes* [60] and can yield cross-protection against other antimicrobial agents, including cefotaxime, cephalothin, ciprofloxacin, and ethidium bromide [61]. More importantly, using one type of disinfectant in certain situations will consistently select the microbes with increasing resistance; therefore, a rationale practice would be to frequently switch to new disinfectants, such as those developed here.

In recent decades, the surge of multiple antimicrobial-resistant pathogens inspired not only the discovery of new antimicrobials but also more effective methods of applying current ones. The synergistic effect of applying multiple antimicrobial components of the same or different types has been proposed. For example, essential oils (permeabilizes cell membrane) can enhance the antimicrobial function of antibiotics and metal nanoparticles to inhibit multi-antibiotics resistant foodborne bacterial pathogens and fungi [62,63,64]. In addition, the combined application of essential oils and other antimicrobials such as gentamicin, amikacin, and ciprofloxacin showed increased inhibition of *P. aeruginosa* [65]. Essential oils loaded onto chitosan nanoparticles also showed strong inhibition against six bacterial species [66]. The superior antimicrobial function of combining antimicrobials, as indicated by numerous studies, inspired us to combine chitosan and ɛ-poly-L-lysine to combat bacterial biofilms.

As natural antimicrobials, both chitosan and ɛ-poly-L-lysine have been extensively studied for their inhibitory effect on microbes. Water-soluble chitosan derivatives can cause membrane permeabilization on bacteria, yeast, and mold [67,68]. Because of its safety, biocompatibility, and biodegradability, chitosan has been tested and applied as a preservative in meat, eggs, vegetables, fruits, and their products [67,69]. In 2013, U.S. Food and Drug Administration conferred GRAS on shrimp-derived chitosan for its application in the food industry [70]. 

ɛ-poly-L-lysine has also received GRAS status from US-FDA [71]. It also inhibited the growth of *E. coli* O157:H7, *S.* Typhimurium, and *L. monocytogenes* in several food products, including beef, rice, and vegetables [72]. Recently, You et al. [73] demonstrated that daily consumption of ɛ-poly-L-lysine for weeks did not cause permanent changes to the gut microbiome in a mouse model, which provides another critical evidence of its safety. Here, we aimed to produce nanoconjugates of the two antimicrobials and test their function specifically in controlling and inactivating bacterial biofilms.

We applied the ionotropic gelation method to synthesize chitosan nanoparticles using a “bottom-up” approach [45,74]. Generally, chitosan molecules are bound to each other with a small linker molecule and form larger gel particles. Chitosan molecules are positively charged when dissolved in a weak acid solution containing TPP forming gel nanoparticles. Not only the ratio of chitosan and TPP, but also the ionic strength, modification of chitosan, pH, and mixing rate affect the size distribution of ChNP [75]. Based on our experience of adapting published conditions for synthesis, minor differences in each laboratory could significantly affect the results; therefore, each parameter should be optimized to produce the ChNP with a desirable dimension. A 3:1 ratio of chitosan and TPP was optimal for the synthesis of ChNP with a median dimension of 150 nm (Figure 1), similar to other reports [45,75]. Further, filtration through a 0.45 µm filter and sonication improved the size distribution of ChNP by removing some undissolved particles. Using a previously established nanoparticles-based inhibition method [28], we determined that 63.7% PL was incorporated in the ChNP-PL matrix (Figure S1).

Chitosan has been applied in various foods as a natural preservative and is highly inhibitory against foodborne pathogens. We compared the MICs of chitosan polymer, and ChNP on five bacterial pathogens, and both showed similar MIC values on *L. monocytogenes*, *S. aureus*, *P. aeruginosa*, *S*. Enteritidis, and *E. coli* (Figure 2). We also counted the viable cells by plating and found that the counts of three bacteria (*P. aeruginosa*, *S.* Enteritidis, and *E. coli* EDL933) in the presence of ChNP were lower than their counts in the presence of chitosan, suggesting ChNP may have superior activity in slowing the growth of certain bacteria at the sublethal concentration. A recent study reported the MIC of chitosan and ChNP to be identical when tested against four bacterial species [45], two (*S. aureus* and *E. coli*) of which were also tested in our study.

Furthermore, we compared the MIC of the mixture of ChNP and PL with each tested separately to determine whether they exhibit synergistic antimicrobial effects. Results on five tested strains clearly showed a synergistic effect (Figure 2) similar to a previous study reported against *Pseudomonas* spp. [41]. This treatment significantly reduced total volatile basic nitrogen formation and extended the shelf life of shrimp without affecting the sensory perception [41]. 

To ensure consistent delivery of two antimicrobials for inactivation of bacteria in biofilms, the ChNP was conjugated with PL (ChNP-PL) and showed a strong inhibitory effect against 19 strains representing species of *Pseudomonas*, *Listeria*, *Salmonella*, *Staphylococcus*, and *E. coli* (Table 1). Furthermore, ChNP-PL also maintained its antimicrobial activity even after 21 days of storage at ambient temperature (Figure 3).

Though research on chitosan as a carrier for drugs, DNA, and peptides have been conducted for decades [76,77,78], the safety of nanoparticulated form still requires a thorough assessment. Various models have been used to determine the safety of both chitosan and PL Huang et al. [79] thoroughly tested the cytotoxicity of chitosan with different molecular weights and chitosan nanoparticles. They reported that both chitosan polymer and ChNP exhibited significant cytotoxic effects on the A549 cell line (lung cancer cell line) when used at a concentration above 0.74 mg/mL, which is much higher than the MIC used in our study. To test the safety of PL, Hiraki et al. (2003) used an absorption, distribution, metabolism, and excretion (ADME) experiment using ^14^C-labeled PL in a rat model and showed that 94% of PL that entered the gastrointestinal tract passed through the feces, and no PL was accumulated in any tissues based on whole-body radiography [80]. This study provided critical evidence as to the foundation for GRAS approval by FDA. We also examined the safety of ChNP-PL by using *in vitro* cell culture (HCT-8) experiment, and ChNP-PL did not induce any cytotoxicity or arrested cell metabolism or cell membrane damage after 13 h of exposure to ChNP (Figure 4). Furthermore, ChNP-PL protected HCT-8 cells from *L. monocytogenes* induced cell damage and maintained the fitness and cellular morphology. 

The inactivation of sessile bacteria in biofilm faces two significant challenges. Firstly, the biofilm matrix, or EPS, is largely made up of polysaccharides, extracellular DNA, and proteins, which provides a dense architecture protecting sessile bacteria from being removed by physical impacts or accessed by large molecules [81,82,83]. Secondly, sessile bacteria globally alter their gene expression, which usually gives them better resistance to antibiotics and several disinfectants [15,84]. The strategies that can be applied to control biofilms in the food processing environment not only have to address these two challenges but also need to consider additional factors. For instance, the applied chemicals could be easily cleaned off, and their residues in food should not raise any safety concerns. Therefore, we were motivated to investigate the potential of using two food-grade molecules, chitosan, and ε-poly-L-lysine, to control the formation of biofilms on food processing or touching surfaces. 

We tested the efficacy of preventing and inactivating the biofilms of five foodborne pathogens. ChNP-PL treatment completely prevented the biofilm formation by *L. monocytogenes*, *P. aeruginosa*, *S*. Enteritidis, and *E. coli* O157:H7, and bacterial counts were undetectable after plating while it partially affected *S. aureus* biofilm formation. ChNP, on the other hand, completely inhibited biofilm formation by *L. monocytogenes* but showed some inhibitory effect against *S. aureus* and *P. aeruginosa*, albeit much lower than ChNP-PL treatment. ChNP treatment surprisingly increased the bacterial counts in biofilms of *S.* Enteritidis 18ENT1344 and *E. coli* EDL933, which suggests a low concentration of ChNP with different surface charge density [85] probably helps promote biofilm formation by these pathogens. It is interesting to note that ChNP in MHB is also inhibitory towards planktonic cells of some *Salmonella* and *E. coli* strains (Table 1). ChNP-PL is also inhibitory towards these pathogens in mixed culture biofilm and prevented the biofilm formation by *L. monocytogenes* when cocultured with *P. aeruginosa* and *S. aureus*, and it also completely inhibited the growth of *P. aeruginosa* but partially inhibited *S. aureus* (Figure 6). These data indicate ChNP-PL is highly effective in preventing biofilm formation by mono- or multi-pathogens. 

We also tested the inactivation of preformed biofilm by ChNP-PL and data show ChNP-PL was highly effective in eliminating monoculture biofilms of *L. monocytogenes*, *S*. Enteritidis, *E. coli* O157:H7 and moderately effective against *S. aureus* but not against *P. aeruginosa* (Figure 7). In the mixed culture biofilm, ChNP-PL is inhibitory towards *L. monocytogenes* and moderately towards *S. aureus* but none towards *P. aeruginosa*. These data indicate *Pseudomonas* being a strong biofilm former protected itself from the lethal effects of ChNP-PL by producing hydrophobic and viscous extracellular matrix [86,87] that prevents ChNP-PL access to the cell membrane. 

## 5. Conclusions

Chitosan nanoparticles (ChNP) of ~100 nm were synthesized using the ionic gelation method, and they were successfully conjugated with ε poly-L-lysin (PL). The ChNP-PL exhibited synergistic antimicrobial activity against all tested pathogens. Furthermore, the ChNP-PL maintained its antimicrobial activity at least for 16–21 days when stored in an ambient condition. ChNP-PL was found to be nontoxic and did not affect cell proliferation when tested against a human intestinal epithelial HCT-8 cell line. ChNP-PL is highly effective in preventing biofilm formation by monocultures of *L. monocytogenes*, *P. aeruginosa*, *S*. *enterica* ser. Enteritidis, and *E. coli* O157:H7 and mixed cultures of *L. monocytogenes* and *P. aeruginosa* or *L. monocytogenes* and *S. aureus*. Likewise, it also inactivated preformed monoculture biofilms of *L. monocytogenes*, *S*. *enterica* ser. Enteritidis, *E. coli* O157:H7 and moderately effective against *S. aureus* but not against *P. aeruginosa.* In the mixed culture biofilm, ChNP-PL is inhibitory towards *L. monocytogenes* and moderately towards *S. aureus* but none towards *P. aeruginosa*. These results show the combination of two natural antimicrobials ε-poly-L-lysin conjugated chitosan nanoparticles have great potential to prevent and disrupt polymicrobial biofilms of foodborne pathogens in food processing facilities.

## Figures and Tables

**Table 1:** Comparison of Minimal Inhibitory Concentration (MIC) values of ChNP and ChNP-PL.

Bacteria	MIC (µg/mL)	
	ChNP	ChNP-PL
*Pseudomonas aeruginosa* ATCC10145	>37.5	12.5–25
*P. putida* PRI107	>37.5	12.5–25
*P. aeruginosa* PRI99	25–37.5	2.5–3.75
*Listeria ivanovii* ATCC19119	25–37.5	2.5–3.75
*L. seeligeri* ATCC 35967	25–37.5	2.5–3.75
*L. marthii* ATCC BAA-1595	25–37.5	2.5–3.75
*L. monocytogenes* F40	25–37.5	2.5–3.75
*L. monocytogenes* F4244	25–37.5	1.25–2.5
*Salmonella enterica* serovar Enteritidis PT21	>37.5	3.75–12.5
*S. enterica* ser. Typhimurium ST1	>37.5	12.5–25
*S. enterica* ser. Heidelberg 18ENT1418	>37.5	12.5–25
*S. enterica* ser. Enteritidis 18ENT1344	>37.5	3.75–12.5
*Staphylococcus aureus* NRRL B767	>37.5	3.75–12.5
*S. aureus* ATCC25923	25–37.5	2.5–3.75
*S. aureus* ATCC29213	25–37.5	2.5–3.75
*Escherichia coli* K12	>37.5	2.5–3.75
*E. coli* O157:H7 SEA13A72	>37.5	2.5–3.75
*E. coli* O157:H7 PT23	>37.5	1.25–2.5
*E. coli* O157:H7 EDL933	>37.5	2.5–3.75

**Table 2:** Bacterial counts in mixed-culture biofilms.

Bacteria	Avg CFU/mL			Fold-Change *
	Initial Inoculum	Monoculture Biofilm	Mixed Culture Biofilm	
*L. monocytogenes* F4244	3.5 × 10^6^	4.1 × 10^8^	2.1 × 10^9^	5-fold ↑
*S. aureus* ATCC25923	7.4 × 10^6^	4.7 × 10^9^	4.2 × 10^8^	10-fold ↓
*L. monocytogenes* F4244	3.5 × 10^6^	4.1 × 10^8^	4.7 × 10^9^	11-fold ↑
*P. aeruginosa* PRI99	7.0 × 10^6^	7.3 × 10^9^	4.6 × 10^9^	1.6-fold ↓
*L. monocytogenes* F4244	3.3 × 10^6^	3.2 × 10^8^	1.1 × 10^8^	2.9-fold ↓
*S. aureus* 2747	1.2 × 10^7^	3.0 × 10^8^	2.1 × 10^8^	1.4-fold ↓
*L. monocytogenes* F4244	3.6 × 10^6^	4.2 × 10^8^	5.1 × 10^7^	8.4-fold ↓
*S. enterica* serovar Enteritidis 1344	6.4 × 10^6^	7.5 × 10^7^	4.0 × 10^7^	1.9-fold ↓
*L. monocytogenes* F4244	3.6 × 10^6^	4.2 × 10^8^	1.4 × 10^7^	30-fold ↓
*E. coli* O157:H7 EDL933	3.4 × 10^6^	7.6 × 10^7^	3.4 × 10^8^	4.5-fold ↑

* ↑ indicate fold increase or ↓-fold decrease relative to initial inoculum

**Table 3:** Prevention of biofilm formation by chitosan nanoparticles.

Bacteria	Avg CFU/cm^2^		
	Untreated	ChNP *	ChNP-PL *
*L. monocytogenes*	9.2 × 10^6^	<50 (>184,000-fold ↓)	<50 (>184,000-fold ↓)
*S. aureus*	8.7 × 10^7^	8.9 × 10^6^ (9.8-fold ↓)	3.6 × 10^4^ (2400-fold ↓)
*P. aeruginosa*	1.7 × 10^7^	5.0 × 10^5^ (34-fold ↓)	<50 (>184,000-fold ↓)
*S. enterica* ser. Enteritidis	1.1 × 10^7^	4.4 × 10^7^ (4-fold ↑)	103 (110,000-fold ↓)
*E. coli* O157:H7	2.7 × 10^7^	1.2 × 10^8^ (4.4-fold ↑)	<50 (>184,000-fold ↓)
**Lm + Sa mixed biofilms**			
*L. monocytogenes*	1.9 × 10^7^	<50 (>184,000-fold ↓)	<50 (>184,000-fold ↓)
*S. aureus*	4.2 × 10^6^	3.8 × 10^6^ (1.1-fold ↓)	1.0 × 10^4^ (420-fold ↓)
**Lm + Pa mixed biofilms**			
*L. monocytogenes*	8.2 × 10^7^	<50 (>184,000-fold ↓)	<50 (>184,000-fold ↓)
*P. aeruginosa*	4.0 × 10^7^	1.0 × 10^3^ (40,000-fold ↓)	<50 (>184,000-fold ↓)

* ↑- fold increase or ↓-fold decrease in bacterial counts relative to untreated control.

## Data Availability

All data are included in the manuscript.

## Supplementary Materials

The following supporting information can be downloaded at: https://www.mdpi.com/article/10.3390/foods11040569/s1, Figure S1. Synthesis and characterization of chitosan nanoparticles (ChNP) and chitosan nanoparticles with ε-poly-L-lysine (ChNP-PL). (a) Zetasizer measurement of ChNP synthesized with sodium tripolyphosphate TPP (Ch:TPP 3:1). Zetasizer measurement of ChNP after filtration through 0.45 μm syringe filters (b) and after 10 cycles of 30 s sonication (c). (d) Zetasizer measurement of ChNP-PL synthesized with 2% PL with or without sonication. (e) Estimation of binding of PL to ChNP based on the inhibitory effect of different concentrations of free PL on *L. monocytogenes* lawn compared to ChNP-PL conjugate on BHI agar plates (See Figure 1b). Figure S2. Analysis of minimum inhibitory concentrations (MICs) of ChNP and ChNP-PL on 19 strains of *L. monocytogenes*, *S. aureus*, *P. aeruginosa*, *S. enterica*, and *E. coli*.
