# Supplementary material for: Inactivation of Polymicrobial Biofilms of Foodborne Pathogens Using Epsilon Poly-L-Lysin Conjugated Chitosan Nanoparticles
Source: Foods. 2022 Feb 16;11(4):569. doi: 10.3390/foods11040569 (PMC8871342; doi:10.3390/foods11040569)
Supplement: Supplementary file 1 [file foods-11-00569-s001.zip › foods-1562277-supplementary.pdf]

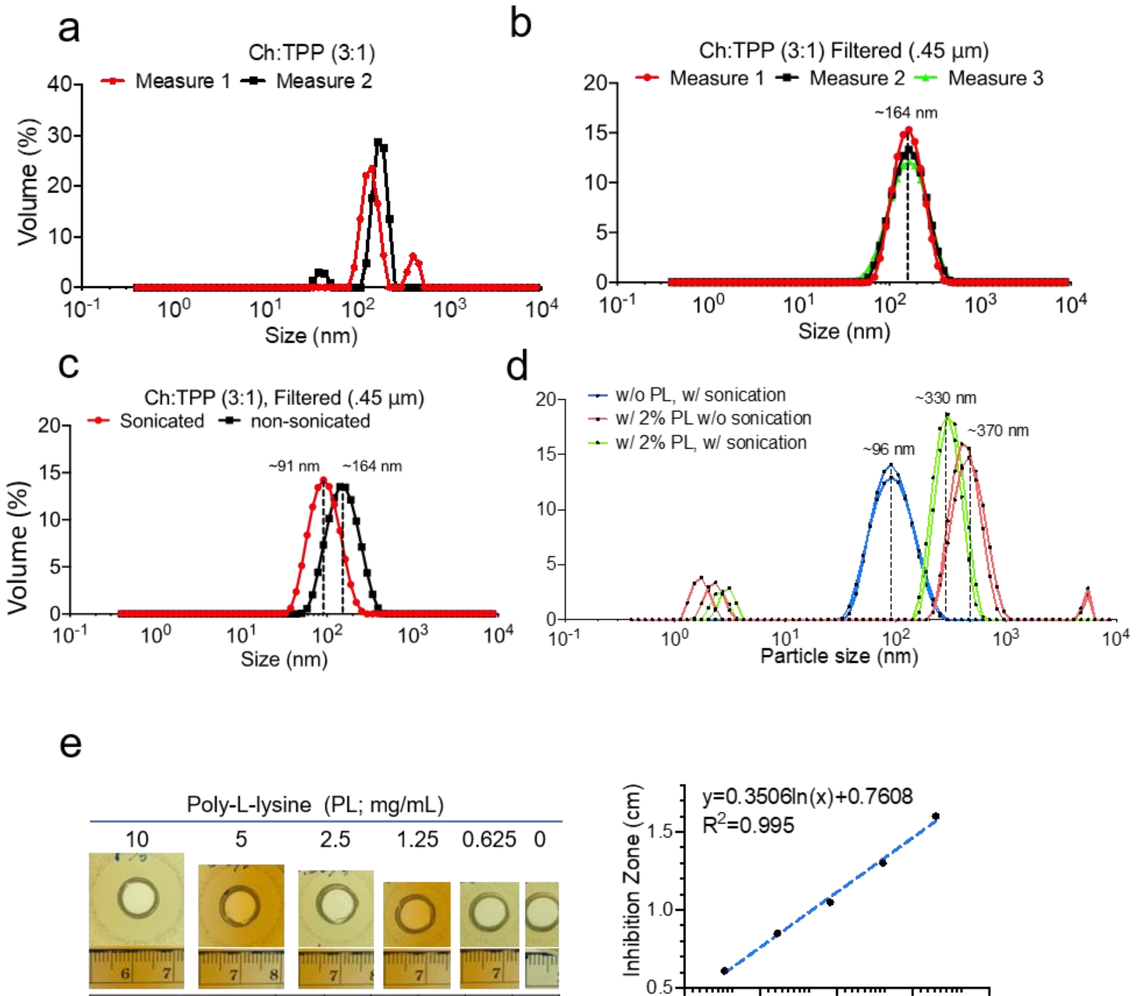

PL Conc (mg/mL)	10	5	2.5	1.25	0.625	0
Total Inhibition Zone (cm)	2.35	2.05	1.8	1.6	1.36	0.75
Active Zone (Total-0.75 cm)	1.6	1.3	1.05	0.85	0.61	

Supplementary Figure S1. Synthesis and characterization of chitosan nanoparticles (ChNP) and chitosan nanoparticles with ε-poly-L-Lysine (ChNP-PL). (a) Zetasizer measurement of ChNP synthesized with sodium tripolyphosphate TPP (Ch:TPP 3:1). Zetasizer measurement of ChNP after filtration through 0.45 µm syringe filters (b) and after 10 cycles of 30s sonication (c). (d) Zetasizer measurement of ChNP-PL synthesized with 2% PL with or without sonication. (e) Estimation of binding of PL to ChNP based on the inhibitory effect of different concentrations of free PL on *L. monocytogenes* lawn compared to ChNP-PL conjugate on BHI agar plates (See Figure 1b).

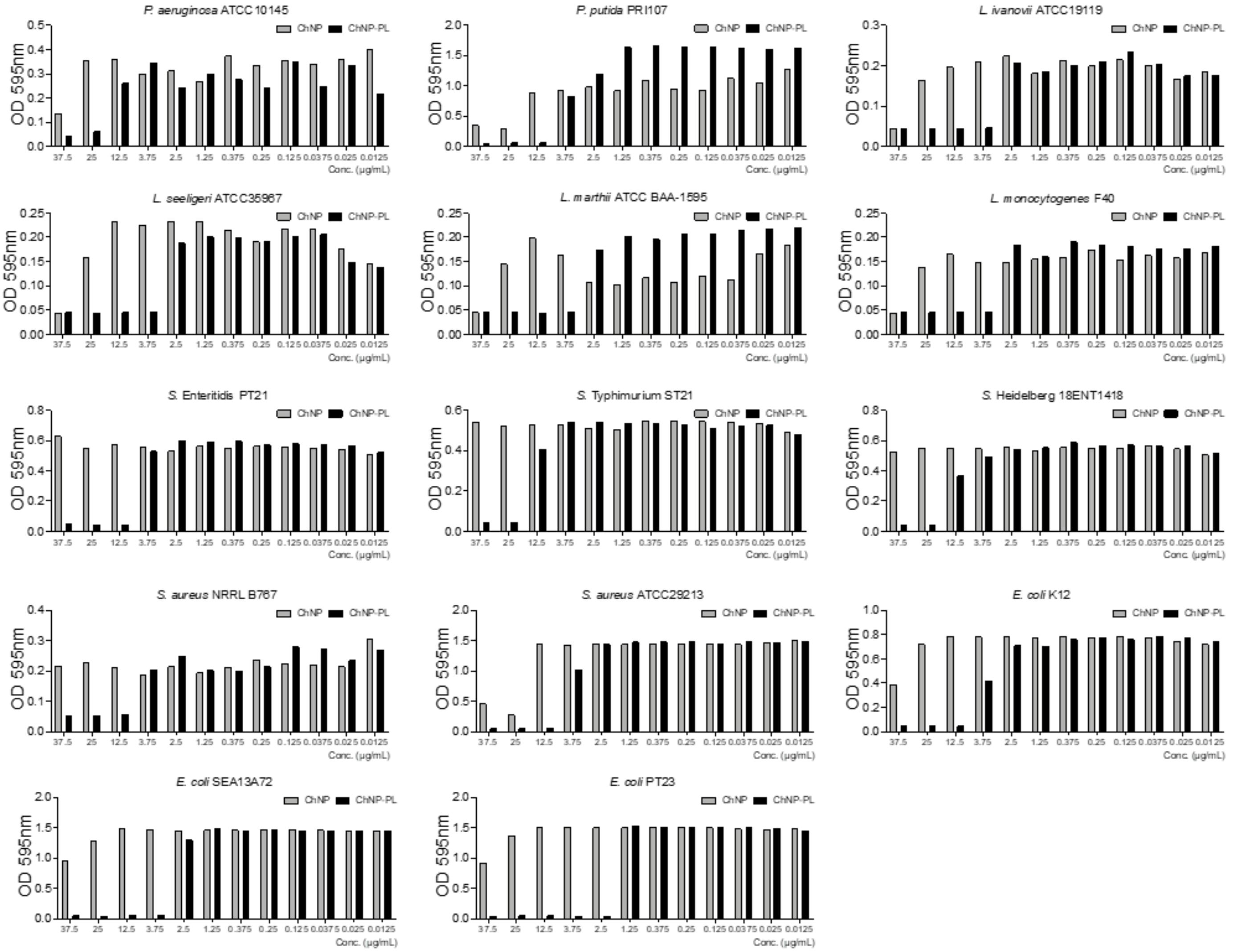

Supplementary Figure S2. Analysis of minimum inhibitory concentrations (MICs) of ChNP and ChNP-PL on 19 strains of *L. monocytogenes*, *S. aureus*, *P. aeruginosa*, *S. enterica*, and *E. coli*.
